# *Lactobacillus* Bacteria in Breast Milk

**DOI:** 10.3390/nu12123783

**Published:** 2020-12-10

**Authors:** Katarzyna Łubiech, Magdalena Twarużek

**Affiliations:** Department of Physiology and Toxicology, Faculty of Biological Sciences, Kazimierz Wielki University, Chodkiewicza 30 St., 85-064 Bydgoszcz, Poland; twarmag@ukw.edu.pl

**Keywords:** breast milk, probiotic microflora, *Lactobacillus* spp., infant nutrition, breastfeeding

## Abstract

Breast milk is an optimal food for infants and toddlers. The composition of breast milk adapts to the needs of the developing organism, satisfying nutritional needs at an early stage of growth and development. The results of research to date have shown that breast milk is the best food for a child, containing not only nutrients but also biologically active substances that aid in the optimal, proper growth and development of infants. Among the many components of breast milk, an important element is the probiotic microflora, including bacteria of the genus *Lactobacillus* spp. These organisms exert a multidirectional, health-promoting effect on the body of children who consume breast milk. The number of lactic acid bacteria, including *Lactobacillus*, colonizing the breast milk environment and their species diversity varies and depends on many factors, both maternal and environmental. Breast milk, as a recommended food for infants, is an important source of probiotic microflora. The aim of this study was to present the current understanding of probiotic bacteria of the genus *Lactobacillus* present in breast milk.

## 1. Introduction

Breast milk is a natural food for infants and young children. It satisfies the nutritional needs of a growing and developing infants on many levels. The World Health Organization (WHO) recommends exclusive breastfeeding for the first six months of a child’s life and continued breastfeeding while expanding the diet at least until the age of two [1]. The American Academy of Pediatrics (AAP) recommends that infants should be breastfed for the first six months of life with continued breastfeeding while expanding their diet with complementary foods for a year or more [2]. The decision on the duration of breastfeeding while expanding a child’s diet considers the individual needs of the mother and child [3]. In 2013–2018, only 41% of children under 6 months of age were exclusively breastfed worldwide [4]. This value represents the worldwide average. The age of children at weaning in developing countries is significantly higher than that in highly developed countries [5]. The benefits of breastfeeding are taken into account in policies of many countries. The promotion of breastfeeding is recognized as an important element of social policy aimed at reducing the mortality of newborns and young children as well as reducing the financial costs of the treatment of children from this age group. The factor that significantly affects the health of breastfed babies is the microbiota of breast milk.

## 2. Materials and Methods

The methodology to perform this review included the following processes: definition of the aim of the review, a literature search, data collection, evaluation, comparison and analysis.

The literature review included electronic searches of Ebsco, Web of Science, Pubmed and ScienceDirect (November 2000–July 2019). A bibliographic search strategy was conducted to identify studies reporting on the microbiota of breast milk especially *Lactobacillus* bacteria. The electronic search used the following terms: breast milk, breast milk microbiota, breastfeeding, recommendations for breastfeeding, variability in breast milk composition, breast milk ingredients, colostrums, transitional milk, mature milk, probiotic microflora of breast milk, *Lactobacillus* spp., *Bifidobacterium*, human milk oligosaccharides, infant nutrition. Reference lists of the previous reviews and relevant studies were examined. Inclusion criteria were papers written in English and Polish published from 1 January 2004 to November 2020. The quality of controlled studies was critically appraised.

## 3. Variability in Breast Milk Composition

Breast milk is perfectly matched to the needs of a growing baby. The composition of breast milk is variable, both in the daily cycle and throughout the entire lactation period [6]. The variability of milk composition is also important due to factors such as the state of health of the mother and breastfed child. Changes in the composition of breast milk ensure the optimal supply of nutrients and biologically active ingredients, depending on current nutritional needs [7]. Breast milk covers not only demand on nutritional ingredients but also provide adequate supply of bioactive nutrients to protect the child. These changes correspondent with current needs of the body [8]. Colostrum appears first after delivery. It is characterized by a very high content of immunoglobulins, primarily secretory immunoglobulin A (sIgA), which support newborn immunity [9]. Immunoglobulins transferred in breast milk are resistant to digestion in the baby’s digestive tract and are specifically adapted to antigens that the child is most likely to come into contact with [10]. Colostrum also contains a high concentration of leukocytes and growth factors such as epidermal growth factor [11]. Compared to milk secreted in subsequent stages of lactation, colostrum contains more protein and less fat and lactose. It is a rich source of micro- and macroelements as well as fat-soluble vitamins [12]. The next stages of lactation are the secretion of transitional milk followed by mature milk. Transitional milk occurs after a period of colostrum secretion that lasts from several days to two weeks after delivery. It still has some of the features of colostrum; however, it is important to increase the amount of milk secreted, which corresponds to the nutritional needs of the newborn. Mature milk is milk secreted from 4–6 weeks after delivery [6].

It is difficult to investigate the effect of the mother’s diet on the composition of breast milk. The conducted studies did not show any correlation between the diversity of the mother’s diet and differences in the composition of breast milk. This may be related to the compensatory mechanisms responsible for maintaining the relative stability of the macronutrient composition in relation to differences in the diet of breastfeeding women. However, there is a relationship between the macronutrient content in human milk and the nutritional value of maternal daily consumption [13]. Although the variability in the maternal diet has no effect on the quantitative fat content of breast milk, an effect on the qualitative composition of fatty acids is observed [14]. Fatty acids act as immunomodulators in breastfed babies [15]. Studies in animal models have shown that they can influence the composition of the intestinal microflora in early life [16]. There is a relationship between the composition of the mother’s body and the composition of the macronutrients of breast milk. There is a positive relationship between the fat content of the mother’s body and the protein content of breast milk [14].

## 4. Probiotic Microflora of Breast Milk

Breast milk is an extremely important source of biologically valuable ingredients, including the probiotic microbiota [17,18,19,20]. The colonization of the organism begins in prenatal life. Many species of bacteria have been identified in neonatal meconium, placenta, and amniotic fluid. The composition of the fetal microbiota may be influenced by maternal factors such as maternal conditions, eating habits, and body weight [21]. Prenatal stress has also been shown to influence the differentiation of the child’s microbiome [22]. It has been investigated that the composition of the microbiome of the first-pass meconium is influenced by maternal factors during pregnancy [23]. The digestive tract of growing children is colonized by microorganisms that largely depend on the type of delivery [24]. The colonization of the child’s body continues during their passage through the mother’s vaginal canal, skin-to-skin contact, and breastfeeding. In addition, some bacterial strains come from the hospital environment. In the case of delivery via cesarean section, colonization with undesirable hospital microorganisms is dominant. Breast milk is one of the main factors that shapes the microbiological balance of the newborn’s digestive tract [25,26]. This is due to the high content of probiotic bacteria [27]. Human milk contains a wide spectrum of bacteria such as *Staphylococci, Streptococci, Corynebacteria,* lactic acid bacteria, *Propionibacteria*, and *Bifidobacteria* [28]. Among these populations, probiotic bacteria are present in an amount of 10^1^–10^7^ colony forming units per mL [29].

There are fewer cases of infection among breastfed children, which may be associated with differences in the composition of the intestinal microbiota [30]. The correct composition of the intestinal microbiota affects the proper functioning of the entire body and prevents the development of many diseases. The benefits of microbial homeostasis are both short- and long-term. An adequate supply of probiotic microorganisms with food supports the proper formation of the microbiological profile and provides maximum benefits from microbiological homeostasis in the gastrointestinal tract [31]. Probiotic microorganisms affect the maturation and development of the immune system [32,33]. In addition, they have trophic and metabolic effects. They affect the integrity of the gastrointestinal mucosa and the production of sIgA antibodies. They also contribute to the formation of the immune system associated with the gastrointestinal mucosa, gut-associated lymphoid tissue (GALT) [34]. The probiotic microbiota is also important for preventing gastrointestinal infections by eliminating or reducing the number of pathogenic microflora. Mechanisms that drive this effect include antimicrobial properties, altering intestinal conditions, and competing for intestinal epithelial adhesion sites and nutrients with pathogens [35]. The stimulation of intestinal mucus production and mobilization of the immune system provides additional protection against intestinal colonization by pathogens [36]. Studies have shown that bacteria present in breast milk may play a role in defense against the pathogenic microorganism *Staphylococcus aureus* [37]. The ability to inhibit the growth of pathogenic bacteria may be due to competitive exclusion and the production of antimicrobial compounds such as bacteriocins, organic acids or hydrogen peroxide [38]. The probiotic microflora also reduces the risk of diabetes, obesity, hypertension and hypercholesterolemia [2]. The microbiological balance of the digestive tract in infants reduces the risk of necrotizing enterocolitis (NEC). NEC is a disease that often requires surgical treatment. The cause of this disease is an immaturity gastrointestinal tract, blood supply disorders, artificial nutrition and disorders of the composition of the intestinal microflora. An efficiently functioning intestinal microflora reduces the risk of diseases such as recurrent streptococcal tonsillitis in children, otitis media, ulcerative colitis, urinary tract and gastrointestinal infections, respiratory tract infections and diarrhea [37]. Maintaining the microbial balance is also important for the proper functioning of the digestive tract. The role of commensal microorganisms involves the ability to break down toxins and carcinogens, synthesis of trace substances, fermentation of undigested food ingredients, and participation in the process electrolyte and mineral salt absorption [34].

In pre-term infants, differences in the composition of the gut microbiota are observed compared to full-term infants. Each stage of pregnancy is important for the proper development of the body. Premature birth results in disturbances in the formation of the optimal composition of the intestinal microbiota, increasing the predisposition to develop disorders such as necrotizing enterocolitis (NEC). The composition of the microbiota of premature babies is dominated by Enterobacteriaceae and *Clostridium*. In term infants *Bifidobacterium*, *Lactobacillus* and *Streptococcus* dominate. Factors disturbing the proper colonization of the intestines include: low birth weight, physiological immaturity, the use of antibiotics and prolonged hospitalization [39]. Breastfeeding is often difficult in cases of pre-term labor. It often takes a great deal of effort and time for the mother to develop sufficient lactation to cover the baby’s nutritional needs. For premature infants, breast milk has therapeutic value. Through the bioactive factors it affects the maturation of the immune system, supports the formation of a proper intestinal microbiome, and protects against infections [40]. Human milk oligosaccharides (HMO) concentration in breast milk of mothers who gave birth prematurely is much higher (2.6 g/dL) compared to the milk of mothers who gave birth on time (0.5–1 g/dL). It may be an adaptation of milk composition to the needs of premature babies, protecting them against infectious diseases to which they are exposed [41]. When breastfeeding is not possible, it is recommended to introduce breast milk derived from donors. For safety reasons, breast milk is pasteurized. This procedure reduces the content of valuable bioactive ingredients. It has been shown that the inoculation of pasteurized donor milk with the addition of mother’s breast milk has a positive effect on the reconstruction of the breast milk microbiota [42].

Breast milk contains not only probiotic microorganisms that affect the composition of the intestinal microbiota but also factors supporting its growth. These factors are oligosaccharides that act as prebiotics [43]. HMOs include more than 200 structurally distinct oligosaccharides [44]. HMOs are prebiotics that support the development of probiotic microflora. Oligosaccharides are not digested in the intestines, which makes them an ideal food for microorganisms, enabling their growth [45]. Qualitative HMO composition varies between women. One of the beneficial effects of HMOs in the digestive tract is their ability to block the binding of pathogens to intestinal epithelial cells. Another anti-pathogenic effect is supporting a population of probiotic intestinal bacteria such as *Bifidobacteria*. HMOs affect the epithelial cells of the intestinal mucosa by modulating apoptosis, proliferation and differentiation [46]. Human milk oligosaccharides have the ability to modulate the immune system. Disturbances of the homeostasis between the host immune system and microbiota modulates inflammation. Immune-suppressive mechanisms are indispensable for intestinal homeostasis. Due to the diversity of these components in human milk, their effect on the infant’s immune system may differ from mother to infant [47].

Breast milk contains non-protein nitrogen in the form of, among other nucleotides, creatinine, free amino acids and peptides. Nucleotides have a beneficial effect on the development of the intestinal microbiota.

The quantity and qualitative composition of the probiotic microflora in breast milk is variable. Factors that determine the content of probiotic bacteria in breast milk can generally be divided into environmental factors and maternal factors. These include the diet during pregnancy and lactation, the mother’s state of health, antibiotic therapy, environmental conditions in which a woman lives, etc. These factors can significantly contribute to a significant diversity in the microbiological composition of milk [48,49]. Research conducted in mice (Reven 2015) showed a relationship between the supply of probiotics to pregnant and lactating mice and the increased detection of the mammary gland microbiota [50]. This confirms that the individual variability of the microbiota composition depends on additional factors, such as probiotic supplementation. A child that consumes approximately 800 mL of breast milk a day absorbs 8 × 10^4^–8 × 10^6^ commensal bacteria [37]. The digestive tract of breastfed children contains a narrow spectrum of Gram-positive bacteria [51]. Only the expansion of the child’s diet to include solid foods significantly increases the species diversity of the intestinal microflora [26,52]. In the breast milk of healthy women, there are commensal bacteria such as *Lactobacilli*, *Lactococci*, *Enterococci* and *Leuconostoc* spp. Among *Lactobacilli* present in breast milk, the following have been identified: *Lactobacillus gasseri*, *Lactobacillus rhamnosus*, *Lactobacillus plantarum*, *Lactobacillus fermentum* and *Enterococcus faecium*. Lactic acid bacteria and *Bifidobacterium* make up to 85% of the entire bacterial population of the intestinal microflora. The human digestive tract contains a complex population of microorganisms. The composition of the microbiota stabilizes between 3 and 5 years of age [31].

## 5. Shaping the Child’s Microbiota

During childbirth, children come into contact with microorganisms that are then involved in shaping the microbiota of their body. The formation of the microbiological profile of the child’s digestive tract is shaped by many factors, the most important of which are the type of delivery, feeding method and environmental factors [53]. This process begins during pregnancy. Scientific reports have confirmed that the amniotic fluid is not microbiologically sterile and that the fetus is already in contact with microorganisms at this stage [54,55]. Childbirth is essential for the colonization of a child’s body by microbes. Natural childbirth is the most beneficial. In this case, the acquisition of the microbiota takes place during the passage through the mother’s genital tract [56]. In addition, the mother’s body is stressed during labor, which causes the mobilization and migration of microorganisms in the woman’s body. The situation is different when a cesarean section is performed. The child is not able to acquire microbiota from the mother’s genital tract, and the first microorganisms that he contacts after delivery are the adverse hospital microflora. A Cesarean section, although needed in many cases and saving the life of a newborn, unfortunately does not provide him with an optimal start. The adverse conditions of children who are born via cesarean section are common knowledge, various actions are undertaken to ameliorate them. One method is to place sterile swabs in the genital tract of a woman giving birth by cesarean section. After delivery, these swabs are used to wipe the newborn. This action aims to colonize the child’s body with the natural microbiota of the mother, even when natural delivery is not possible [57].

Even children who were born via cesarean section have the opportunity to significantly improve the probiotic microflora profile by consuming mother’s milk. Exclusive breastfeeding is essential in this case. The microbiological profile of the gastrointestinal tract of children exclusively fed with breast milk is different from children fed with formula or in a mixed manner. *Lactobacilli* counts are higher in breastfed infants than formula-fed infants [58]. Changes that occur after the administration of formula may be somewhat offset by subsequent breastfeeding, but some differences in the species diversity of microorganisms that inhabit the baby’s digestive tract remain that cannot be changed. That is why breastfeeding immediately after delivery is so important. The microbiological profile of the digestive tract of newborns who use formula promotes the development of allergic reactions, autoimmune diseases, and many other disease entities, including civilization diseases. Studies of breastfed children have shown that they have a lower susceptibility to NEC, a disease that is extremely dangerous in the first stage of life, especially among premature babies. The microbiota of breast milk affects the microbiota of the child’s digestive tract, and thus is an important factor in maintaining body homeostasis, preventing many disease entities in both the short and long term [59]. The conducted studies indicate the possibility of influencing the composition of breast milk by taking probiotics by pregnant and lactating women. A relationship was observed between maternal supplementation with multi-component probiotic preparations and IL6 mean values in colostrum and between IL10 and TGF-β1 mean values in mature breast milk. Changes in the composition of breast milk resulted in a reduced incidence of infantile colic and regurgitation in infants [60]. Studies on the effect of probiotic supplementation by pregnant and lactating women showed a relationship between the amount of *Lactobacilli* and *Bifidobacteria* in the colostrum and mature milk of mothers receiving probiotic preparation with vaginal delivery compared to mothers receiving placebo. There was no difference in women who had a caesarean section [61]. Also, research conducted by Abrahamsson et al. have shown the ability to transfer the *Lactobacillus* bacteria in breast milk after oral supplementation of women in the final stages of pregnancy [62].

## 6. The Origin of Bacteria in Breast Milk

The origin of bacteria in breast milk is not entirely clear. One of the sources of microorganisms found in breast milk is the digestive tract of the mother—the bacterial entero-mammary pathway [17,28,63]. The migration of microorganisms in the gastrointestinal tract occurs via the mesenteric lymph nodes and then dendritic cells [64]. Dendritic cells (DCs) can penetrate the intestinal epithelium and thus can take up bacteria from inside the intestines [64]. DCs can take up intestinal-derived bacteria without disrupting the integrity of the intestinal epithelial barrier. The opening of tight junctions between intestinal epithelial cells is possible due to the expression of tightly bound proteins [17]. These proteins include occludin, claudin 1 and Junctional Adhesion Molecule (JAM). They can create junction-like structures with surrounding epithelial cells [65]. The transport mechanism of non-pathogenic microorganisms also applies to CD18+ cells, including macrophages. After penetrating DCs or macrophages, the intestinal bacteria can move to other places, including the mammary gland during the lactation period [17]. Microorganisms pass further into the lumen of secretory vesicles in the mammary gland, and together with milk, enter the baby’s body, where they participate in the formation of the intestinal microbiota of the newborn. There is also a theory that microorganisms enter breast milk through the skin of the nipple. Bacteria can exist on the surface of the nipple and in the mammary duct system in the form of a biofilm [26] and provide a source of microorganisms for the suckling child [66,67,68]. Each of these sources plays a role in shaping the specific microbiota of breast milk. A study showed that the milk of women who directly breastfeed children has a greater proportion of bacteria of the phylum *Actinobacteria* and family *Veillonellaceae* compared to pumped breast milk [69]. This confirms that some of the microorganisms in the breast milk environment come from the mouth of a breastfeeding child. The newborn’s oral cavity is inhabited by microorganisms originating from the female reproductive tract during delivery. It was proven that children fed pumped breast milk achieved a higher body mass index later in life than children who were fed breast milk directly from the breast [70,71]. This may be due to the lack of microorganisms from the child’s mouth in the microbiota of pumped breast milk. In addition, the method of collecting milk and its storage can significantly change components that have important effects on the child’s health. Breastfeeding directly from the mother’s breast may affect the regulation of the baby’s food intake.

It has been suggested that neonates can acquire vaginal strains of probiotic microorganisms during labor and then transmit these bacteria to the breast during breastfeeding [68]. Research has indicated that the mother’s vaginal microflora has a small effect on the qualitative composition of the microbiota of mother’s milk and infant feces. However, a correlation was found between the composition of the milk microbiota and the infant’s feces, which indicates a strong role of breastfeeding in the formation of the intestinal microbiome [25].

Studies have shown the presence of DNA and cellular structures of intestinal bacteria in the placenta, amniotic fluid and fetal membranes [54,55,66]. This means that the baby is in an environment where microorganisms are present even before delivery, and these may also colonize the baby’s body. Scientists have concluded that all these mechanisms contribute to the colonization of the mammary gland with probiotic microorganisms.

## 7. Bacteria of the Genus *Lactobacillus* spp.

There are more than 200 strains of bacteria in breast milk, of which the most important are *Lactobacilli, Bacteroides* and *Bifidobacterium*. *Lactobacillus* bacteria (belonging to lactic acid bacteria) have the ability to break down lactose and other simple sugars into lactic acid [72]. The methodology of research on the presence of *Lactobacillus* bacteria in breast milk includes breeding studies on the Man, Rogosa, and Sharpe (MRS) agar medium. Some researchers use MRS supplemented with l-cysteine (0.5 g/L) (MRS-Cys) Incubation is usually carried out in the conditions of: 37 °C for 48–72 h. Identification at the species level can be performed by mass spectrometry MALDI (Matrix-assisted laser desorption/ionization) or by polymerase chain reaction (PCR) sequencing.

The microbiological profile of breast milk varies among individuals, both in quality and quantity. Some strains are constantly present in breast milk, while others such as *Lactobacillus* are characterized by variability and are not present in milk samples from every woman [73]. For example, Sinkiewicz and Ljunggren isolated *L. reuteri* from the milk of only approximately 15% of breastfeeding women [74]. Soto et al. detected the presence of *Lactobacillus* bacteria in 40.91% of tested samples. *L. casei*, *L. fermentum*, *L. gasseri*, *L. gastricus*, *L. plantarum*, *L. reuteri*, *L. rhamnosus*, *L. salivarius*, *L. vaginalis*, and L. *rhamnosus* can be isolated from breast milk [25]. *L. salivarius*, *L. fermentum* and *L. gasseri* are dominant in this group. Tests showed a lower frequency of bacteria of the genus *Lactobacillus* in milk samples from women who underwent antibiotic therapy or cesarean section. A relationship was also found between the reduced detection of *L. salivarius* and the use of anesthesia during delivery [75]. The spectrum of *Lactobacillus* groups is narrow in breast milk and infant feces. The composition of bacterial strains is specific and includes only a small number of *Lactobacilli* strains [25].

Human milk is an important source of lactic acid bacteria in shaping the intestinal microflora of breast-fed infants. A study by Matsumiya et al. showed that less than one-fourth of newborns with vaginal delivery were acquired by vaginally derived lactic acid bacteria. After one month, these bacteria were replaced with lactic acid bacteria from breast milk [76]. *Lactobacillus* composition is similar within a mother-child pair and contains small number of *Lactobacilli* strains [37]. Studies indicate that lactic acid bacteria in the gastrointestinal tract are found only in 74% of infants in the first months of life. Ahrné et al. isolated one strain of lactic acid bacteria from 37% of infants, 26% had two strains, and 11% had three or more strains. In this study, most babies were fed breast milk. Lactobacillus bacteria were more often isolated from the stools of infants receiving breast-milk than from weaned infants [77]. Research by Martin et al. indicate the presence of the same specific *Lactobacillus* strains in breast milk and breastfed infant faeces [78].

Compared to that in formula-fed infants, the intestinal microbiota of breastfed infants is richer in *Lactobacilli* and *Bifidobacteria* [58]. At the same time, a reduced number of *Veillonellaceae*, *Enterococcaceae*, *Streptococcaceae* and *Lachnospiraceae* is observed [79]. Many studies confirm the health-promoting effects of *Lactobacillus* bacteria derived from breast milk on the baby’s body. Studies have shown the effectiveness of disease prevention by *L. reuteri, L. rhamnosus* and many others [80,81]. The probiotic potential of *Lactobacillus* bacteria isolated from breast milk is similar to that of strains used in probiotic products used in medicine [82,83]. A beneficial effect of *Lactobacillus* bacteria in the prevention and treatment of mastitis in women was also found [84]. *Lactobacillus* strains isolated from breast milk may be included in the treatment of mastitis. Their safety is appreciated due to their natural origin, anti-infectious and immunomodulatory properties [85].

Studies have shown that the oral intake of *Lactobacillus* bacteria, previously isolated from breast milk, effectively reduces mastitis during lactation caused by *Staphylococcus aureus*. This could be due to the ability of *Lactobacilli* to adhere to the surface of epithelial cells, inhibit adhering pathogens and promote mucin production [86].

The oral intake of *Lactobacillus* strains by breastfeeding women results in the presence of these strains in human milk samples and their isolation from the feces of breastfed children. Various *Lactobacillus* strains differentially modulate the immune system, e.g., *L. fermentum* has an immunomodulatory effect, while *L. salivarius* has an anti-inflammatory effect [87].

Table 1 presents research on Lactobacillus bacteria in breast milk and important key results.

## 8. Summary and Conclusions

Probiotic bacteria are an important element that contributes to the proper development of children. Many studies have reported the beneficial effect of probiotic bacteria, including *Lactobacillus* bacteria, in breast milk on the formation of the infant’s intestinal microbiome. It is important to understand factors that may influence the shaping of the microbiological profile of both breast milk and the child’s digestive tract and their mutual correlations. As probiotic bacteria in the diet has great benefits, it is important to ensure the optimal quality of food consumed by children, including breast milk as the first recommended food for children from birth. Research has indicated that various factors influence the composition of milk, including the qualitative composition of the human milk microbiome. Species diversity is influenced by external factors, but it is also regulated by endogenous factors of the female body. Many factors can affect the human milk microbiota. These factors can generally be divided into those derived from the environment and maternal factors. These include the lactation period, maternal dietary habits and nutritional status as well as mode of delivery, gestational age. Also, external factors like geographical location, and the use of antibiotics or other medicines can have an influence on the milk microbiota composition [88]. *Lactobacillus* bacteria are less often isolated from breast milk samples of women who had received antibiotherapy during pregnancy or lactation [75]. Dietary habits, the environment and social conditions may contribute to a significant differentiation of the composition of breast milk microbiota [49]. The conducted studies indicate a lower species diversity of human milk microbiota from women who underwent cesarean section. Higher levels of *Staphylococcus* spp. and lower levels of *Enterococcus* spp. are detected in breast milk derived from mothers who had C-section compared with vaginal deliveries. The *Bifidobacterium* group is detected more frequently in vaginal than in C-section deliveries. This difference is not statistically significant [89]. Detection of *Lactobacilli* is higher in breast milk of women who have vaginally delivered than women who have delivered by caesarean section [75].

The mother’s health and medical conditions may affect the composition of the milk microflora. For example, the milk of allergic mothers contains a lower number of *Bifidobacterium longum* compared to non-allergic mothers [90]. Mastitis is another factor influencing the composition of breast milk. Breast milk of women suffering from mastitis is dominated by *Staphylococcus aureus* [91]. Breast milk of women with celiac disease has reduced levels of *Bacteroides* spp. and *Bifidobacterium* spp. [92]. Indicate the influence of acquired immune deficiency syndrome (AIDS) on the breast milk composition. Breast milk samples derived from HIV-positive women has higher bacterial diversity and higher prevalence of *Lactobacillus* spp. than those of non-HIV-positive women [93].

The mother’s body weight may also influence the composition of the breast milk microbiota. The number of *Bifidobacterium* bacteria was lower, while the number of *Staphylococcus* bacteria was higher in the breast milk of overweight mothers than in normal-weight mothers [94].

Probiotic bacteria of the genus *Lactobacillus* show multidirectional pro-health effects on the human body. Their presence in breast milk seems to be extremely valuable in shaping the correct microbiota of breastfed infants. It is particularly important to provide probiotic strains to premature babies. The results of the research indicate that strains of bacteria of the genus *Lactobacillus* are isolated from breast milk samples only in some of the breastfeeding women and their species differentiation in the breast milk of individual women is very narrow. Typically, one strain belonging to the genus *Lactobacillus* is isolated. It is of great importance to recognize the factors that may modulate the quantitative and qualitative composition of the breast milk microbiota.

## Figures and Tables

**Table 1:** Research on *Lactobacillus* in breast milk and important key results.

Results:	Source:
*Lactobacilli* isolated from 40.91% samples of breast milk *Bifidobacteria* isolated from 10.61% samples of breast milk Isolated species: • *L. salivarius* isolated from 13.64% of samples • *L. fermentum* isolated from 10.61% of samples • *L. gasseri* isolated from 9.09% of samples • *L. reuteri* isolated from 11.88% samples • *L. plantarum* isolated from 10.63% samples • *L. rhamnosus* isolated from 8.13% samples • *L. casei* isolated from 4.38% samples	Soto et al. 2014 [75]
*L. reuteri* isolated from the breast milk of only approximately 15% of breastfeeding women	Sinkiewicz et al. 2008 [74]
*Lactobacilli* counts higher in breastfed infants gut microbiota than formula-fed infants	Rinne et al. 2005 [58] Ahrné et al. 2005 [77]
A positive relationship between the amount of *Lactobacilli* and *Bifidobacteria* in the colostrum and mature milk of mothers receiving probiotic preparation with vaginal delivery compared to mothers receiving placebo, no difference in women who had a caesarean section	Mastromarino et al. 2015 [61]
Ability to transfer the *Lactobacillus* bacteria in breast milk after oral supplementation of women in the final stages of pregnancy	Abrahamsson et al. 2009 [62]
Individual variability of *Lactobacillus* strains in breast milk	Jost et al. 2013 [73]
*Lactobacillus* composition is similar within a mother-child pair and contains small number of *Lactobacilli* strains	Heikkilä et al. 2003 [37] Martín et al. 2012 [78]
*Lactobacillus* strains isolated from breast milk may be included in the treatment of mastitis	Arroyo et al. 2010 [85]
*Lactobacillus* strains differentially modulate the immune system, e.g., *L. fermentum* has an immunomodulatory effect, while *L. salivarius* has an anti-inflammatory effect	Díaz-Ropero et al. 2007 [87]
